# Effect of hydroalcoholic extract of *ginger* on the liver of epileptic female rats treated with lamotrigine

**Published:** 2014

**Authors:** Ameneh Poorrostami, Farah Farokhi, Reza Heidari

**Affiliations:** 1 *Department of Biology,Faculty of science,Urmia university, I. R. Iran*

**Keywords:** *Epilepsy*, *Lamotrigine*, *Liver*, *Zingiber officinale*, *Rat*

## Abstract

**Objective**: Lamotrigine is an antiepileptic drug, widely used in the treatment of epilepsy; long-term use of this drug can cause hepatotoxicity. *Zingiber officinale* Roscoe *(ginger*) possesses antioxidant properties. In present research, the effect ofhydroalcoholic extract of *ginger* (HEG) on the liver of lamotrigine-treated epileptic rats was investigated

**Material and Methods:** Forty-eight female Wistar rats were selected and allocated to 8 groups of 6 each. Group 1: Negative controls were treated with normal saline. Group 2: Positive controls were treated with lamotrigine (LTG) (10 mg/kg) daily by gavages for 4 consecutive weeks. Epilepsy was induced in treatment groups by i.p. injection of pentylenetetrazol (PTZ) (40 mg/kg). Group 3: Epileptic group received normal saline (10 ml/kg). Group 4: Epileptic group was treated with LTG (10 mg/kg). Groups 5 and 6: Epileptic groups received HEG (50 and 100 mg/kg). Groups 7 and 8: Epileptic groups received LTG and HEG (50 and 100 mg/kg). At the end of 28 days, blood samples were drawn and their livers were processed for light microscopy.

**Results: **The mean values of TG, CHOL, AST, and ALT activity significantly rose (p<0.01) in groups 2, 3, and 4, while in rats treated with HEG (groups 5, 6, 7, and 8), the levels of liver enzymes significantly decreased (p<0.05) compared with epileptic group treated with lamotrigine (group 4). Histopathological changes of liver samples were comparable with respective control.

**Conclusion: **These results suggest that hydroalcoholic extract of *ginger* improves liver function in lamotrigine-induced hepatotoxicity.

## Introduction

Chemical kindling is an experimental model of epilepsy and epileptogenesis in which repeated application of initially subconvulsive chemical stimulation induces progressive seizure activity (Mason and Cooper, 1972 ▶).

Pentylenetetrazol (PTZ)-induced kindling is associated with a variety of behavioral, neurophysiological, and neurochemical alterations resulting in long-lasting changes in hippocampal and cortical glutamate receptor density (Schröder et al., 1993 ▶).

Lamotrigine (LTG) is antiepileptic drug, widely used in the treatment of epilepsy and bipolar disorder. It is a phenyl triazine derivative and its mechanism of action is related to the blockade of voltage-dependent sodium channels which stabilises presynaptic membranes and inhibits excitatory neurotransmitter release (Messenheimer, 1995 ▶). Patients receiving chronic treatment with lamotrigine in the form of single or polytherapy are at a high risk of developing signs and symptoms of drug toxicity. A case of fatal progressive hepatotoxicity in a patient treated with LTG was reported (Leach et al., 1986 ▶; Overstreet et al., 2002 ▶). 

Liver is vulnerable to drug-induced toxicity mainly because of its role as a primary organ of drug elimination and its subsequent exposure to potential toxins. Many commonly prescribed medications including virtually all of the major antiepileptic drugs can cause hepatotoxicity. Hepatic reactions to LTG ranged from transient elevation of hepatic enzymes without clinical signs or symptoms of hepatic dysfunctions to fatal hepatotoxicity (Overstreet et al., 2002 ▶; Meshkibaf et al., 1995 ▶; Betts et al., 1991 ▶).

Oxidative stress, resulting from an imbalance in the generation of free radicals and antioxidant defense molecules, affects biological macromolecules causing their structural alterations that lead to cell damage and its death (Ryter et al., 2007 ▶). Long-term use of certain antiepileptic drugs has also been shown to increase oxidative stress (Maertens et al., 1995 ▶). The most important effect of free radicals is lipid peroxidation, which causes disruption of cell membrane thereby leading to their destruction (Barber and Bernheim, 1967 ▶). It is reported that free radical generation due to the increased activity of the glutamatergic transmitter plays a crucial role in neuronal cell death of the PTZ kindling in rats (Rocha et al., 1996 ▶; Schroder et al., 1993 ▶; Sechi et al., 1997 ▶; Sejima et al., 1997 ▶; Rauca et al., 1999 ▶; Becker et al., 1997 ▶).

Botanical medicines have been used traditionally by herbalists and indigenous healers worldwide for the prevention and treatment of liver disease (Takeoka and Dao, 2003 ▶). *Ginger *is gaining popularity amongst modern physicians and its underground rhizomes are the medicinally useful part (Mascolo et al., 1989 ▶). The important active components of the *ginger* root are thought to be volatile oils and pungent phenol compounds such as gingerols, shogaols, zingerone, and gingerols (Sekiwa et al., 2000 ▶; Zancer et al., 2002 ▶). The pharmacological effects of *ginger* and its pungent constituents, fresh and dried rhizome were investigated. Among the demonstrated effects, anti-platelet, antioxidant, anti-tumor, antirhinoviral, anti-hepatotoxicity, anti-arthritic, and antidiabetic effects can be mentioned (Fisher-Rasmussen et al., 1991 ▶; Sharma et al., 1994 ▶; Kamtchoving et al., 2002 ▶; Islam and Choi, 2008 ▶).

*Ginger* was found to have hypocholesterolemic effects and causes decrease in body weight, glucose in blood, serum total cholesterol, and serum alkaline phosphatase in adult male rats (Bhandari et al., 2005 ▶). *Ginger* extract attenuated, in a dose-dependent manner, CCl4 and acetaminophen-induced increases in the activities of ALT, AST, and ALP in rats blood (Yemitan and Izegbu, 2006 ▶). Other investigators have also shown the hypolipidemic effect of *ginger* (sharma et al., 1996 ▶). Akhani et al. (2004) ▶ reported that* ginger* treatment significantly decrease both serum cholesterol and triglycerides. The study of Gujaral revealed that serum and liver cholesterol decreased when *ginger *was administered to hypercholesterolemic rats (Gujaral et al., 1978 ▶). Gingerol inhibited lipid peroxidation induced by FeCl3-ascorbate system (Aeschbach et al., 1994 ▶). Gingerol inhibited the oxidative activity of xanthine which generated reactive oxygen species (ROS), for example superoxide anions (Chang et al., 1994 ▶).In this research, protective effect of hydroalcoholic extract of *ginger* against lamotrigine-induced hepatotoxicity was investigated.

## Materials and Methods

**Plant materials**

The *ginger* rhizomes (*Z. officinale*) was purchased from the local market and identified by a professor from the Department of Biology at Urmia University with herbarium number of 4G041. The rhizomes was peeled, chopped into tiny bits, air-dried for 2 weeks, and ground with a mechanical grinder. The ground plant (500 g) was macerated in 70% ethanol for 48 h, filtered with a white cloth and the filtrate was concentrated using a rotary evaporator at an optimum temperature of 40-50 °C (Anosike et al., 2009 ▶). The dried yield of the extract was 5 g. 

**Experimental animals**

Forty-eight adult female rats weighing 200-230 g (obtained from the Pasteur Institute central animal house, Tehran, Iran) were used for the study. They were fed with standard diet pellets and allowed food and water *ad-libitum* for an acclimation period of 4 weeks. The animals were maintained in a strictly controlled temperature (22-25 ^o^C). Twelve hours of light and dark cycles were followed in a fully ventilated room.

**PTZ kindling**

Over a period of 20 days, animals were injected intraperitoneally with subconvulsive doses of PTZ (40 mg/kg) in saline every 48 h. After each injection, the convulsive behavior was observed for 30 min, and antiseizure results were scored as follows: stage 0, no response; stage 1, ear and facial twitching; stage 2, convulsive waves axially through the body; stage 3, myoclonic jerks and rearing; stage 4, clonic convulsions with the animal falling on its side; and stage 5, repeated severe tonic–clonic convulsions or lethal convulsions. The animals were considered to be kindled after having received 10 PTZ injections and reached at least three consecutive stage 4 or stage 5 seizures (Becker et al., 1995 ▶).

**Chemicals**

Pentylenetetrazole was obtained from sigma chemicals company, Germany. Kits of ALT, AST, cholesterol, and triglycerides were purchased from ZiestChem Diagnostics, Tehran, Iran. Lamotrigine was obtained from BakhtarBioshimicompany (Tehran, Iran). 

**Experimental protocol**

The experimental animals were divided into 8 groups, each group contained 6 animals: Group 1: Negative controls were treated with normal saline (C1). Group 2: Positive controls were treated with LTG (10 mg/kg) daily by gavages for 4 consecutive weeks (CL2). Epilepsy was induced in treatment groups by i.p. injection of PTZ (40 mg/kg). Group 3: Epileptic group received normal saline (10 ml/kg) (CP3). Group 4: Epileptic group were treated with LTG (10 mg/kg) (LP4). Groups 5 and 6: Epileptic groups (PG5, PG6) received HEG (50 and 100 mg/kg, respectively). Groups 7 and 8: Epileptic groups (LPG7, LPG8) received LTG and HEG (50 and 100 mg/kg, respectively) daily by gavages for 4 weeks.

**Preparation of liver homogenate**

At the end of study, livers were immediately removed and weighed. Each liver was cut longitudinally into two halves; one half was fixed in 10% phosphate-buffered formalin for histological examination, while the other half was stored at – 70 °C for biochemical analysis (Magda et al., 2011 ▶).

**Catalase (CAT) activity measurement**

Catalase activity was measured following the method of Aebi (1984) ▶ Phosphate buffer (0.50 mM, pH 7.0) and 30% H2O2 were freshly prepared. Two ml of sample solution was mixed with 1 ml of H_2_O_2_, and the decomposition of hydrogen peroxide was measured spectrophotometrically at 240 nm against a blank containing 2 ml of sample solution and 1 ml of phosphate buffer. To avoid the intervention by bubbling, the reaction time was controlled by not exceeding 30 seconds**.**

**Determination of lipid peroxidation (MDA)**

Liver lipid peroxidation was determined by the formation of thiobarbituric acid reactive substances (TBARS) according to the method of Ledwozyw et al. (1986) ▶. Malondialdehyde (MDA) is formed as an end product of lipid peroxidation which reacts with TBA reagent under acidic condition to generate a pink colored product. Liver suspension (0.1 ml) was added to 0.4 ml of distilled water, followed by the addition of 2.5 ml of trichloroacetic acid (TCA) and left at room temperature for 15 minutes. TBA (1.5 ml) was then added and heated in a water bath at 100 °C for 30 minutes until a faint pink color was obtained. After cooling, the color was extracted in 1 ml of butanol and the intensity was measured using the spectrophotometer at 535 nm.

**Statistical analysis**

All values were expressed as mean±SEM and the differences were compared using (ANOVA) followed by Tukey´s multiple comparison tests. For all analyses, p<0.05 were considered significant.

## Results

**Histological Findings**

**Figure 1:**
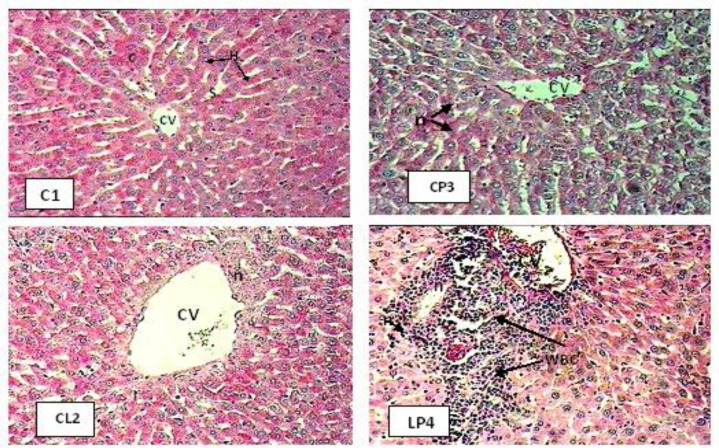
The effects of lamotrigine and PTZ on hepatic tissues of rats stained with Periodic Acid Schiff (PAS, ×400). Normal central vein (CV), Hepatocyte cells (H), sinusoids (S), carbohydrates (C), Hepatocyte necrosis (n), white blood cells (WBC).

Histological studies of control animals showed normal hepatocyteswith normal storage of carbohydrates (Figure 1). (C1) The liver section of LTG treated animals (Group 2) (CL2) showed extension of central vein, hepatocyte necrosis around central vein, and decrease in carbohydrates, whereas the liver section of PTZ–treated animals (Group 3) (CP3) showed intense centrilobular, necrosis and vacuolization and decreased storage of carbohydrates. 

The livers of animals treated with PTZ and LTG (Group 4) (LP4) showed congestion of central vein, hepatocyte necrosis, presence of white blood cells (WBC), and decrease in carbohydrates. The animals treated with different doses of HEG showed recovering of hepatocyte, especially in the 100 mg/kg group, with minimal inflammation and near-normal architecture showing higher protective activity as compared with other groups. However, the liver sections of rats on treatment with hydroalcoholic extract of *ginger* exhibited significant liver protection, as evident by the presence of normal hepatic cords, absence of necrosis, and increasing carbohydrates storage, when compared with the standard drug and control animals.

**Figure 2:**
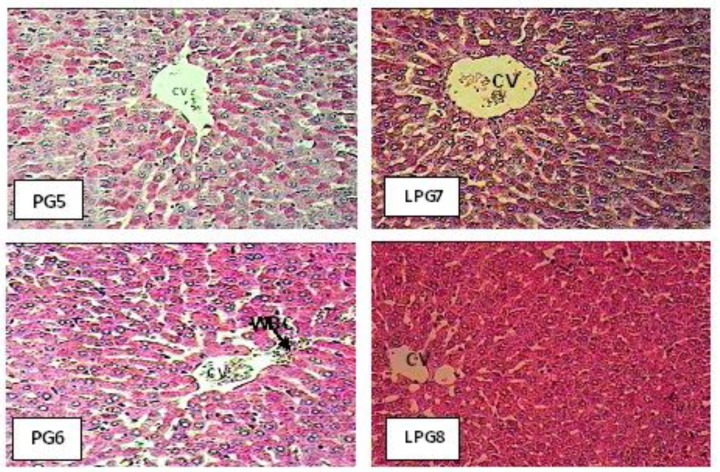
The effects of lamotrigine, PTZ, and *ginger* on rats hepatic tissue stained with Periodic Acid Schiff (PAS, X400). Normal central vein (CV), white blood cells (WBC).

Histological studies of difference epileptic groups treated showed increase storage of carbohydrates (Figure 2). PG5: Liver section of PTZ group treated with hydroalcoholicextract of *ginger* (50 mg/kg) showed increased storage of carbohydratesand extension of central vein**. **PG6: Specimen obtained from epileptic groups treated with HEG (100 mg/kg), showed increased storage of carbohydrates compared with LP group and presence of white blood cells (WBC) in sinusoids. LPG7: Liver section of epileptic groups treated with lamotrigine and HEG (50 mg/kg) demonstrated increased storage of carbohydrates and extension of central vein compared with PG5. LPG8: Liver section of epileptic groups treated with lamotrigine and HEG (100 mg/kg) exhibited increased storage of carbohydrates with normal central vein (CV) and hepatocyte cells**.**

**Biochemical findings**

Table 1 shows that the mean values of liver enzymes (ALT and AST) in all of the experimental groups of rats were significantly increased when compared with normal rats (C1). Treatment of the epileptic rats with 100 mg/kg HEG (PG6 and LPG8) caused a significant reduction in the serum ALT and AST (p<0.05) when compared with epileptic group treated with lamotrigine.

Similarly, the serum cholesterol and triglyceride levels were significantly higher in all of the experimental groups compared with normal rats (C1). However, treatment of the epileptic rats with 100 mg/kg HEG (PG6 and LPG8) caused a significant reduction in the serum cholesterol and triglyceride levels (p<0.05) when compared with epileptic group treated with lamotrigine.

Table 2 shows that the mean value of malondialdehyde in all the experimental groups of rats was significantly increased when compared with normal rats (C1). The obtained results in this research showed that malondialdehyde levels were significantly raised in epileptic rats (Group 3) (p<0.01) as compared with the negative control rats. Treatment of the epileptic rats with 100 mg/kg HEG (PG6 and LPG8) caused a significant reduction in MDA level (p<0.05) when compared with epileptic group treated with lamotrigine. 

Treatment of the epileptic rats with 100 mg/kg HEG (PG6 and LPG8) caused a significant increase in CAT activity (p<0.05) when compared with epileptic group treated with lamotrigine. 

**Table 1:** Serum levels of liver enzymes and plasma lipids profile of the experimental groups

**Groups**	**C**_1_	**CL**_2_	**CP**_3_	**LP**_4_	**PG**_5_	**PG**_6_	**LPG**_7_	**LPG**_8_
**ALT(U/L)** **mean ±SEM**	31.53±0.46	66.49±0.45	64.52 ±0.26	73.1±0.17	62.4±0.36 +	56.01±0.41 +	60.1±0.15 +	54.4±0.35 +
**AST(U/L)** **mean ±SEM**	84.13±0.41	118.52±0.54	116.06±0.4	120.29±0.67	101.13±0.19 +	97.35±0.5 +	98.79±0.38 +	93.69±0.51 +
**Cholesterol (mg/dl)** **mean ±SEM**	111.26±0.4	134.3±0.34	132.20±0.37	136.3±0.32	128.76±0.2 +	123.35±0.23 +	126.6±0.3 +	119.27±0.3 +
**Triglyceride** **(mg/dl)** **mean ±SEM**	70.77±0.42	124.58±0.34	117.08±0.19	130.25±0.51	86.61±0.55 +	82.52±0.26 +	84.67±0.36 +	80.54±0.27 +

Values are expressed as mean±SEM, n=8. Statistical difference between different experimental groups *vs.* negative control (Group 1)

**: p<0.01; Statistical difference between different experimental groups *vs.* epileptic group treated with lamotrigine (Group 4) +: p<0.05. C1: Negative control, CL2: Positive control, CP3: PTZ control, LP4: LTG+PTZ_, _PG5: PTZ+HEG50, PG6: PTZ+HEG100, LPG7: LTG+PTZ+HEG50, LPG8: LTG+PTZ+HEG100.

**Table 2:** Levels of malondialdehyde and catalase in liver tissue of the experimental groups

**Groups**	**C**_1_	**CL**_2_	**CP**_3_	**LP**_4_	**PG**_5_	**PG**_6_	**LPG**_7 _	**LPG**_8_
**MDA(n mol/g tissue)** **mean ±SEM**	30.7±1.08	59.03±0.15**	56.23±0.67**	63.83±0.8	52.53±0.38 +	46.6±0.47 +	50.23±0.5 +	41.06±1.03 +
**CAT(U/mg protein)** **mean ±SEM**	146.8±0.85	84.2±0.55	89.2±0.15	80.6±0.62	111.06±1.56 +	128.53±0.49 +	116.06±0.86 +	131.03±1.28 +

Values are expressed as mean±SEM, n=8. Statistical difference between different experimental groups *vs.* negative control (Group 1)

**: p<0.01; Statistical difference between different experimental groups *vs.* epileptic group treated with lamotrigine (Group 4) +: p<0.05. C1: Negative control, CL2: Positive control, CP3: PTZ control, LP4: LTG+PTZ_, _PG5: PTZ+HEG50, PG6: PTZ+HEG100, LPG7: LTG+PTZ+HEG50, LPG8: LTG+PTZ+HEG100.

## Discussion

Liver is the main organ responsible for drug metabolism and appears to be the sensitive target site for substances modulating biotrans formation (Gram and Gillette, 1971 ▶). Clinical squeal of hepatic failure in a patient treated with lamotrigine were reported. The liver damage was documented in serial liver biopsies, which showed approximately 50% hepatocyte necrosis (Overstreet et al., 2002 ▶). In this research liver section of rats treated with lamotrigen showed extension of central vein, decreased storage of carbohydrates, and hepatocyte necrosis.

Specific reason to question a causal relationship between LTG therapy and hepatotoxicity was provided by a recent study in which a cohort study conducted by means of prescription-event monitoring (PEM) reported LTG to be safe when used for refractory epilepsy but there were individual cases of hepatotoxicity (Mackay et al., 1997 ▶). The mechanism of lamotrigine-induced liver failure that occurred is not clearly understood. Previous reports have attributed this reaction to an immune-mediated allergic reaction (Fayad et al., 2000 ▶; Mecarelli et al., 2005 ▶; Overstreet et al., 2002 ▶; Sauve et al., 2000 ▶). Histopathological findings in this research revealed the presence of white blood cells (WBC) around central vein in Group 4 (Figure 1, LP4). 

Free radicals and reactive oxygen species (ROS) are continuously produced in the body. These oxygen species are the cause of cell damage and the progression of tumor cells to cancer cells. Therefore, tissues must be protected from oxidative injury through intracellular (SOD, GPx, and catalase) as well as extracellular (vitamins, micronutrients, and antioxidants originated from herbs) antioxidants (Halliwell and Gutteridage, 1999 ▶). Oxygen free radicals are mostly removed by endogenous antioxidants such as superoxide dismutase (SOD), glutathione peroxidase (GPx), and catalase (Das, 2002 ▶). Lipid peroxidation could change the properties of biological membranes, resulting in severe cell damage and thus play a significant role in the pathogenesis of diseases. It has been shown that certain lipid peroxidation products induce fibrogenic cytokines and increases the synthesis of collagen (Mi-Ok and Jeon-Ok, 2010 ▶). 

The oxidative damage in tissue can be limited by exogenous antioxidants. The most important defenses are enzymatic antioxidants, such as SOD, CAT, and non-enzymatic antioxidants as GSH (Wang et al., 2004 ▶). SOD, a manganese-containing enzyme is the first antioxidant enzyme to deal with oxyradicals by accelerating the dismutation of superoxide to hydrogen peroxide, while CAT is a peroxisomalhemeprotein that catalyses the removal of hydrogen peroxide formed during the reaction catalyzed by SOD (Weydert et al., 2006 ▶). Catalase is an enzyme responsible for detoxification of H_2_O_2_ formed by the action of superoxide dismutase. The process of epileptogenesis and long-term use of certain antiepileptic drugs has been shown in previous studies to cause increase in reactive oxygen species leading to oxidative stress and neuronal damage in patients with epilepsy. The activity of the enzyme catalase has been shown to decline in epileptic patients (Sudha et al., 2001 ▶). 

The obtained results in this research indicated that in epileptic rats (Group 3) the catalase activities decreased (p<0.01) when compared with negative control group. The malondialdehyde assay is often considered as an index of free radical generation which increases in conditions of oxidative stress (Kehrer, 1993 ▶). The obtained results from this research showed that the mean value of malondialdehyde levels were significantly raised in treated rats with LTG and PTZ (Table 2). These results confirm the observations of previous studies which had shown increased lipid peroxidation in pentylenetetrazole-induced kindling in rats (Rauca et al., 1999 ▶).

Previous studies indicated that the administration of aqueous extract of *ginger* to rats, orally and intraperitoneally, at two different levels of doses, significantly decreased the activities of some serum enzymes such as aspartate aminotransaminase (AST) and alanine aminotransaminase (ALT) (Alnaqeeb et al., 2003 ▶). *Ginger* and silymarin reduced serum ALT, AST, and ALP indicating membrane stabilization and antioxidant properties of *ginger* (Bhandari et al., 2003 ▶). Results obtained in the present study revealed that the values of serum AST and ALT were significantly decreased in rats treated with LTG and *ginger *100 mg compared with epileptic group treated with lamotrigine. Histophatological changes of liver in these groups were improved and storage of carbohydrates was increased. 

This indicated the effectiveness of *ginger* in prevention of LTG hepatotoxicity. The mechanism of hepatic injury may be due to inflammatory process so the hepatoprotective activity of *ginger* may be due to its content of volatile oils, which showed anti-inflammatory, analgesic, and immunomodulatory effects. Volatile oil of *ginger* is capable of inhibiting T lymphocyte-dependent immune reactions (Zhou et al., 2006 ▶)

The effect of the ethanol extract of the rhizome of *Z. officinale* was tested against carbon tetrachloride and acetaminophen-induced liver toxicities in rats. CCl4 and acetaminophen induced many histopathological changes and increased the activities of ALT, AST, ALP, LDH, and SDH in the blood serum. *Ginger *extract was found to have a protective effect against CCl4 and acetaminophen-induced damage as confirmed by histopathological examination of the liver (Yemitan and Izegbu, 2006 ▶). Chang et al., found the bioactive component of *ginger,* namely gingerol, possessed antioxidative effect by inhibiting peroxidation of phospholipids induced by xanthine oxidase activity. Amin and Hamza demonstrated that *Z. officinal* increased the activities of testicular antioxidant enzymes, superoxide dismutase, glutathione, and catalase and reduced level of malondialdhyde. In this research, treating epileptic animals with LTG+*ginger* (100 mg/kg, Group 8) induced a significant decrease in the malondialdehyde which is lipid peroxidation marker and a significant increase in the level of serum antioxidant enzyme. Siddaraju and Dharmesh reported that *ginger*-free phenolic and *ginger*hydrolysed phenolic fractions exhibited free radical scavenging, inhibition of lipid peroxidation, DNA protection, and reducing power abilities indicating strong antioxidant properities. 

Anti-inflammatory activity of *ginger* is due to the presence of gingerols which have the ability to inhibit prostaglandins and leukotriene synthesis, (Nurtjahja et al., 2003 ▶). In the present study, treating epileptic animals with *ginger* extract (100 mg/kg, Group 8) induced a significant decrease in cholesterol and triglycerides levels. These finding are in agreement with previous studies that treatment with *ginger* juice significantly decreased triglycerides and cholesterol levels in diabetic rats. Reduction in serum lipid levels with *ginger* might be due to its antagonistic action on streptozotocin receptors, thereby increasing insulin levels (Akhani et al., 2004 ▶). The ethanolic extract of *ginger* significantly reduced serum total cholesterol and triglycerides and increased the HDL-cholesterol levels (Bhandari et al., 2005 ▶). Hypolipidemic and anti-atherosclerotic effects of *ginger *extract were also demonstrated in cholesterol-fed rabbits (Bhandari and Grover, 1998 ▶). It was concluded that the hypocholesterolemic effect of *ginger* could have possibly resulted from the inhibition of cellular cholesterol biosynthesis after the consumption of the extract (Fuhrman et al., 2000 ▶).

Hydroalcoholic extract of *ginger* protects liver tissue from lipid peroxidation and exhibits a significant lipid lowering activity in epileptic rats. 
